# 3-Meth­oxy-2-[5-(naphthalen-2-yl)-4,5-di­hydro-1*H*-pyrazol-3-yl]phenol

**DOI:** 10.1107/S1600536814005972

**Published:** 2014-03-22

**Authors:** Dongsoo Koh, Yoongho Lim, Alan J. Lough

**Affiliations:** a Department of Applied Chemistry, Dongduk Women’s University, Seoul 136-714, Republic of Korea; b Division of Bioscience and Biotechnology, BMIC, Konkuk University, Seoul 143-701, Republic of Korea; c Department of Chemistry, University of Toronto, Toronto, Ontario, M5S 3H6, Canada

## Abstract

The asymmetric unit of the title compound, C_20_H_18_N_2_O_2_, contains two independent mol­ecules in which the dihedral angles between the naphthalene ring system [r.m.s. deviations = 0.012 (1) and 0.015 (1) Å] and the benzene ring are 71.65 (6) and 74.51 (6)°. In the crystal, pairs of N—H⋯O hydrogen bonds form two independent inversion dimers with graph-set notation *R*_2_^2^(14). In addition, each mol­ecule contains an intra­molecular O—H⋯N hydrogen bond with an *S*(6) motif.

## Related literature   

For the synthesis and biological properties of pyrazoline derivatives, see: Hwang *et al.* (2013 ▶); Sharifzadeh *et al.* (2013 ▶); Congiu *et al.* (2010 ▶); Khode *et al.* (2009 ▶); Karthikeyan *et al.* (2007 ▶). For related structures, see: Fun *et al.* (2012 ▶); Jasinski *et al.* (2010 ▶). For hydrogen-bond graph-set notation, see: Bernstein *et al.* (1995 ▶).
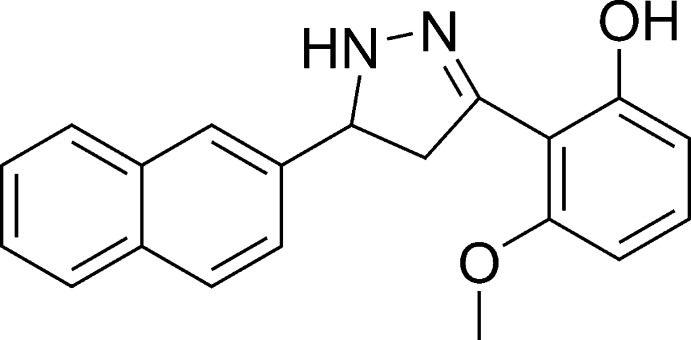


## Experimental   

### 

#### Crystal data  

C_20_H_18_N_2_O_2_
*M* *_r_* = 318.36
Monoclinic, 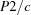

*a* = 21.0215 (15) Å
*b* = 5.6564 (5) Å
*c* = 28.785 (2) Å
β = 110.543 (3)°
*V* = 3205.1 (4) Å^3^
*Z* = 8
Cu *K*α radiation
μ = 0.69 mm^−1^
*T* = 147 K
0.17 × 0.10 × 0.07 mm

#### Data collection  

Bruker Kappa APEX DUO CCD diffractometer
Absorption correction: multi-scan (*SADABS*; Bruker, 2012 ▶) *T*_min_ = 0.695, *T*_max_ = 0.753
43387 measured reflections
5506 independent reflections
4776 reflections with *I* > 2σ(*I*)
*R*_int_ = 0.045

#### Refinement  

*R*[*F* ^2^ > 2σ(*F* ^2^)] = 0.037
*wR*(*F* ^2^) = 0.097
*S* = 1.05
5506 reflections
451 parameters
H atoms treated by a mixture of independent and constrained refinement
Δρ_max_ = 0.17 e Å^−3^
Δρ_min_ = −0.20 e Å^−3^

### 

Data collection: *APEX2* (Bruker, 2012 ▶); cell refinement: *SAINT* (Bruker, 2012 ▶); data reduction: *SAINT*; program(s) used to solve structure: *SHELXS97* (Sheldrick, 2008 ▶); program(s) used to refine structure: *SHELXL97* (Sheldrick, 2008 ▶); molecular graphics: *PLATON* (Spek, 2009 ▶); software used to prepare material for publication: *SHELXTL* (Sheldrick, 2008 ▶).

## Supplementary Material

Crystal structure: contains datablock(s) I. DOI: 10.1107/S1600536814005972/is5346sup1.cif

Structure factors: contains datablock(s) I. DOI: 10.1107/S1600536814005972/is5346Isup2.hkl

Supporting information file. DOI: 10.1107/S1600536814005972/is5346Isup3.cml

CCDC reference: 992239

Additional supporting information:  crystallographic information; 3D view; checkCIF report

## Figures and Tables

**Table 1:** Hydrogen-bond geometry (Å, °)

*D*—H⋯*A*	*D*—H	H⋯*A*	*D*⋯*A*	*D*—H⋯*A*
O1*A*—H1*OA*⋯N1*A*	0.97 (2)	1.71 (2)	2.5754 (15)	145 (2)
O1*B*—H1*OB*⋯N1*B*	0.91 (2)	1.71 (2)	2.5377 (15)	149 (2)
N2*A*—H2*NA*⋯O1*A* ^i^	0.914 (18)	2.234 (18)	3.0470 (16)	147.7 (15)
N2*B*—H2*NB*⋯O1*B* ^ii^	0.911 (19)	2.131 (19)	2.9787 (16)	154.4 (15)

Symmetry codes: (i) 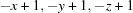; (ii) 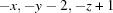.

**Fig. 1.**
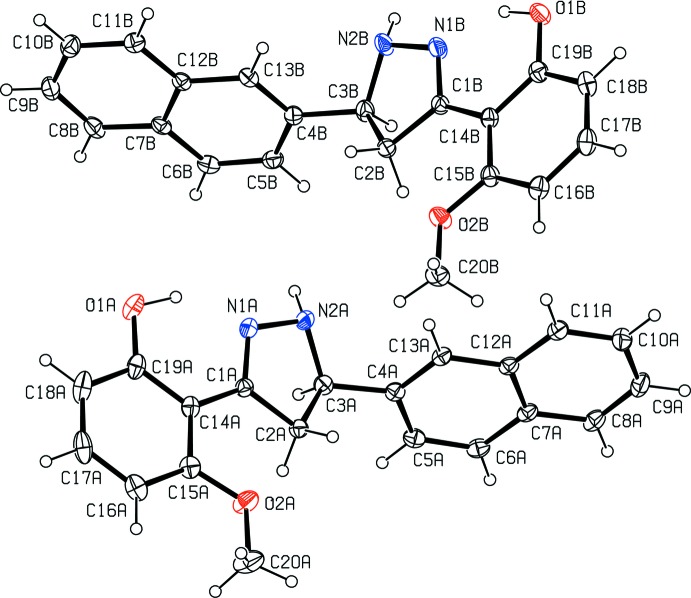
The asymmetric unit of title compound showing 30% probability ellipsoids.

**Fig. 2.**
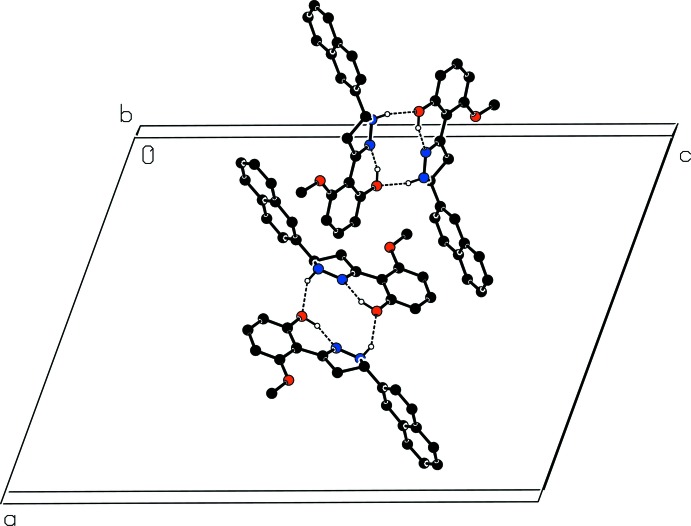
Part of the crystal structure showing hydrogen bonds as dashed lines.

## supplementary crystallographic information

### 1. Comment

Pyrazolines have been reported to show a broad spectrum of biological activities including antibacterial (Sharifzadeh *et al.*, 2013), anticonvulsant (Karthikeyan *et al.*, 2007), analgesic (Khode *et al.*, 2009) and antitumor properties (Congiu *et al.*, 2010). In continuation of our research interest to develop novel pyrazoline derivatives which show broad range of biological activities (Hwang *et al.*, 2013), the title compound (I) was synthesized and its crystal structure was determined.

The asymmetric unit of (I) contains two indepencent molecules (A and B in Fig. 1). The dihedral angles between the naphthalene ring system [r.m.s. deviations 0.012 (1) Å for A and 0.015 (1) Å for B] and the benzene ring are 71.65 (6) and 74.51 (6)° for molecules A and B, respectively. In the crystal, pairs of N—H···O hydrogen bonds form two independent inversion dimers (Fig. 2) with graph-set notations *R*^2^_2_(14) (Bernstein *et al.*, 1995). In addition, each molecule contains an intramolecular O—H···N hydrogen bond with an S(6) notation. Some examples of pyrazoline structures have been published (Fun *et al.*, 2012; Jasinski *et al.*, 2010).

### 2. Experimental

To a solution of 6-methoxy-2-hydroxyacetophenone (10 mmol, 1.66 g) in 50 ml of ethanol was added 2-naphthaldehyde (10 mmol, 1.56 g) and the temperature was adjusted to around 276–277 K in an ice-bath. To the reaction mixture was added 10 ml of 50% (*w*/*v*) aqueous KOH solution and reaction mixture was stirred at room temperature for 60 h. At the end of the reaction, ice water was added to the mixture and acidified with 6 N HCl (pH = 3–4). The resulting precipitate was filtered and washed with water and ethanol. The crude solid was purified by recrystallization from ethanol to give pure chalcone (m.p.; 403–403 K, yield; 63%). Excess hydrazine monohydrate (1 ml of 64–65% solution, 13 mmol) was added to chalcone compound (5 mmol, 1.52 g) in 30 ml anhydrous ethanol, and the solution was refluxed at 363 K for 3 h. The reaction mixture was cooled to room temperature to yield a solid that was then filtered. The crude solids were purified by recrystallization from ethanol to afford pure pyrazolines (m.p.; 403–403 K, yield; 93%). How were the X-ray quality crystals grown. Repeated recrystallization in ethanol gave colourless needle shape crystals suitable for X-ray diffraction.

### 3. Refinement

Hydrogen atoms bonded to C atoms were placed in calculated positions with C—H distances ranging from 0.95–1.00 Å and included in the refinement in a riding-model approximation with *U*_iso_(H) = 1.2*U*_eq_(C) or 1.5*U*_eq_(C_methyl_). H atoms bonded to N and O atoms were refined independently with isotropic displacement parameters.

### Crystal data

**Table:**

C_20_H_18_N_2_O_2_	*F*(000) = 1344
*M**_r_* = 318.36	*D*_x_ = 1.320 Mg m^−^^3^
Monoclinic, *P*2/*c*	Cu *K*α radiation, λ = 1.54178 Å
Hall symbol: -P 2yc	Cell parameters from 857 reflections
*a* = 21.0215 (15) Å	θ = 3.3–56.5°
*b* = 5.6564 (5) Å	µ = 0.69 mm^−^^1^
*c* = 28.785 (2) Å	*T* = 147 K
β = 110.543 (3)°	Needle, colourless
*V* = 3205.1 (4) Å^3^	0.17 × 0.10 × 0.07 mm
*Z* = 8	

### Data collection

**Table:**

Bruker Kappa APEX DUO CCD diffractometer	5506 independent reflections
Radiation source: Bruker ImuS	4776 reflections with *I* > 2σ(*I*)
Multi-layer optics monochromator	*R*_int_ = 0.045
φ and ω scans	θ_max_ = 66.8°, θ_min_ = 2.2°
Absorption correction: multi-scan (*SADABS*; Bruker, 2012)	*h* = −24→23
*T*_min_ = 0.695, *T*_max_ = 0.753	*k* = −6→6
43387 measured reflections	*l* = −33→33

### Refinement

**Table:**

Refinement on *F*^2^	Primary atom site location: structure-invariant direct methods
Least-squares matrix: full	Secondary atom site location: difference Fourier map
*R*[*F*^2^ > 2σ(*F*^2^)] = 0.037	Hydrogen site location: inferred from neighbouring sites
*wR*(*F*^2^) = 0.097	H atoms treated by a mixture of independent and constrained refinement
*S* = 1.05	*w* = 1/[σ^2^(*F*_o_^2^) + (0.0508*P*)^2^ + 0.6182*P*] where *P* = (*F*_o_^2^ + 2*F*_c_^2^)/3
5506 reflections	(Δ/σ)_max_ = 0.001
451 parameters	Δρ_max_ = 0.17 e Å^−^^3^
0 restraints	Δρ_min_ = −0.20 e Å^−^^3^

### Special details

**Table:**

Geometry. All e.s.d.'s (except the e.s.d. in the dihedral angle between two l.s. planes) are estimated using the full covariance matrix. The cell e.s.d.'s are taken into account individually in the estimation of e.s.d.'s in distances, angles and torsion angles; correlations between e.s.d.'s in cell parameters are only used when they are defined by crystal symmetry. An approximate (isotropic) treatment of cell e.s.d.'s is used for estimating e.s.d.'s involving l.s. planes.
Refinement. Refinement of *F*^2^ against ALL reflections. The weighted *R*-factor *wR* and goodness of fit *S* are based on *F*^2^, conventional *R*-factors *R* are based on *F*, with *F* set to zero for negative *F*^2^. The threshold expression of *F*^2^ > σ(*F*^2^) is used only for calculating *R*-factors(gt) *etc*. and is not relevant to the choice of reflections for refinement. *R*-factors based on *F*^2^ are statistically about twice as large as those based on *F*, and *R*- factors based on ALL data will be even larger.

### Fractional atomic coordinates and isotropic or equivalent isotropic displacement parameters (Å^2^)

**Table:**

	*x*	*y*	*z*	*U*_iso_*/*U*_eq_	
O1A	0.49628 (5)	0.5886 (2)	0.56791 (4)	0.0409 (3)	
O2A	0.34244 (6)	1.2274 (2)	0.54722 (4)	0.0440 (3)	
N1A	0.41312 (6)	0.7105 (2)	0.48182 (4)	0.0315 (3)	
N2A	0.38431 (6)	0.7224 (2)	0.42980 (4)	0.0326 (3)	
C1A	0.39789 (6)	0.8982 (2)	0.50121 (5)	0.0246 (3)	
C2A	0.35765 (6)	1.0709 (2)	0.46192 (5)	0.0254 (3)	
H2AA	0.3748	1.2344	0.4698	0.030*	
H2AB	0.3087	1.0666	0.4572	0.030*	
C3A	0.37111 (6)	0.9740 (2)	0.41647 (5)	0.0277 (3)	
H3AA	0.4136	1.0476	0.4149	0.033*	
C4A	0.31402 (6)	1.0150 (2)	0.36773 (5)	0.0252 (3)	
C5A	0.31489 (7)	1.2249 (2)	0.34150 (5)	0.0297 (3)	
H5AA	0.3516	1.3323	0.3544	0.036*	
C6A	0.26385 (7)	1.2767 (2)	0.29782 (5)	0.0304 (3)	
H6AA	0.2657	1.4192	0.2809	0.036*	
C7A	0.20822 (7)	1.1210 (2)	0.27751 (5)	0.0267 (3)	
C8A	0.15504 (7)	1.1653 (3)	0.23166 (5)	0.0335 (3)	
H8AA	0.1560	1.3055	0.2137	0.040*	
C9A	0.10281 (7)	1.0100 (3)	0.21309 (5)	0.0379 (4)	
H9AA	0.0679	1.0421	0.1823	0.045*	
C10A	0.10019 (7)	0.8025 (3)	0.23919 (5)	0.0361 (3)	
H10A	0.0632	0.6961	0.2261	0.043*	
C11A	0.15082 (7)	0.7530 (3)	0.28344 (5)	0.0305 (3)	
H11A	0.1485	0.6122	0.3008	0.037*	
C12A	0.20652 (6)	0.9091 (2)	0.30362 (5)	0.0250 (3)	
C13A	0.26073 (6)	0.8605 (2)	0.34863 (5)	0.0249 (3)	
H13A	0.2602	0.7180	0.3659	0.030*	
C14A	0.42050 (6)	0.9222 (2)	0.55531 (5)	0.0267 (3)	
C15A	0.39484 (7)	1.0994 (3)	0.57825 (5)	0.0312 (3)	
C16A	0.42105 (7)	1.1334 (3)	0.62929 (5)	0.0381 (4)	
H16A	0.4040	1.2561	0.6442	0.046*	
C17A	0.47218 (7)	0.9862 (3)	0.65800 (5)	0.0440 (4)	
H17A	0.4907	1.0107	0.6928	0.053*	
C18A	0.49680 (7)	0.8056 (3)	0.63740 (6)	0.0437 (4)	
H18A	0.5315	0.7049	0.6579	0.052*	
C19A	0.47060 (6)	0.7707 (3)	0.58629 (5)	0.0320 (3)	
C20A	0.31232 (10)	1.4005 (3)	0.56934 (6)	0.0507 (5)	
H20A	0.2743	1.4766	0.5435	0.076*	
H20B	0.3463	1.5199	0.5863	0.076*	
H20C	0.2957	1.3235	0.5934	0.076*	
O1B	−0.09997 (5)	−0.95204 (18)	0.51393 (4)	0.0354 (2)	
O2B	−0.06378 (5)	−0.28470 (19)	0.62119 (4)	0.0374 (3)	
N1B	0.01760 (6)	−0.7754 (2)	0.55421 (5)	0.0369 (3)	
N2B	0.08805 (6)	−0.7481 (2)	0.56768 (5)	0.0386 (3)	
C1B	−0.00720 (6)	−0.6051 (2)	0.57246 (5)	0.0272 (3)	
C2B	0.04742 (6)	−0.4300 (2)	0.59985 (5)	0.0273 (3)	
H2BA	0.0617	−0.4504	0.6362	0.033*	
H2BB	0.0323	−0.2649	0.5911	0.033*	
C3B	0.10441 (6)	−0.4987 (3)	0.58063 (5)	0.0302 (3)	
H3BA	0.0992	−0.4067	0.5498	0.036*	
C4B	0.17604 (6)	−0.4685 (2)	0.61734 (5)	0.0254 (3)	
C5B	0.21368 (7)	−0.2659 (2)	0.61442 (5)	0.0299 (3)	
H5BA	0.1936	−0.1499	0.5897	0.036*	
C6B	0.27874 (7)	−0.2345 (2)	0.64672 (5)	0.0305 (3)	
H6BA	0.3032	−0.0968	0.6441	0.037*	
C7B	0.31015 (6)	−0.4033 (2)	0.68385 (5)	0.0257 (3)	
C8B	0.37838 (7)	−0.3815 (3)	0.71720 (5)	0.0336 (3)	
H8BA	0.4044	−0.2473	0.7151	0.040*	
C9B	0.40695 (7)	−0.5509 (3)	0.75216 (5)	0.0365 (4)	
H9BA	0.4528	−0.5350	0.7737	0.044*	
C10B	0.36893 (7)	−0.7484 (3)	0.75651 (5)	0.0349 (3)	
H10B	0.3887	−0.8631	0.7815	0.042*	
C11B	0.30344 (7)	−0.7755 (3)	0.72472 (5)	0.0294 (3)	
H11B	0.2783	−0.9107	0.7277	0.035*	
C12B	0.27239 (6)	−0.6065 (2)	0.68752 (5)	0.0234 (3)	
C13B	0.20506 (6)	−0.6342 (2)	0.65330 (5)	0.0249 (3)	
H13B	0.1797	−0.7702	0.6554	0.030*	
C14B	−0.07952 (6)	−0.6070 (2)	0.56665 (5)	0.0260 (3)	
C15B	−0.10804 (6)	−0.4417 (3)	0.59036 (5)	0.0289 (3)	
C16B	−0.17715 (7)	−0.4442 (3)	0.58272 (5)	0.0328 (3)	
H16B	−0.1959	−0.3304	0.5985	0.039*	
C17B	−0.21851 (7)	−0.6140 (3)	0.55187 (5)	0.0362 (3)	
H17B	−0.2658	−0.6137	0.5464	0.043*	
C18B	−0.19260 (7)	−0.7822 (3)	0.52909 (5)	0.0344 (3)	
H18B	−0.2215	−0.8983	0.5084	0.041*	
C19B	−0.12339 (7)	−0.7809 (3)	0.53664 (5)	0.0293 (3)	
C20B	−0.09006 (7)	−0.1221 (3)	0.64781 (6)	0.0381 (4)	
H20D	−0.0534	−0.0195	0.6683	0.057*	
H20E	−0.1093	−0.2096	0.6691	0.057*	
H20F	−0.1256	−0.0254	0.6243	0.057*	
H1OA	0.4697 (11)	0.574 (4)	0.5327 (9)	0.078 (7)*	
H1OB	−0.0542 (11)	−0.935 (4)	0.5237 (8)	0.069 (6)*	
H2NA	0.4147 (8)	0.653 (3)	0.4179 (6)	0.043 (5)*	
H2NB	0.1010 (8)	−0.803 (3)	0.5426 (7)	0.049 (5)*	

### Atomic displacement parameters (Å^2^)

**Table:**

	*U*^11^	*U*^22^	*U*^33^	*U*^12^	*U*^13^	*U*^23^
O1A	0.0324 (5)	0.0480 (7)	0.0397 (6)	0.0140 (5)	0.0092 (5)	0.0120 (5)
O2A	0.0597 (7)	0.0477 (7)	0.0276 (5)	0.0247 (6)	0.0192 (5)	0.0042 (5)
N1A	0.0282 (6)	0.0323 (7)	0.0286 (6)	0.0055 (5)	0.0032 (5)	−0.0009 (5)
N2A	0.0313 (6)	0.0335 (7)	0.0279 (6)	0.0108 (5)	0.0041 (5)	−0.0042 (5)
C1A	0.0209 (6)	0.0250 (7)	0.0276 (7)	−0.0002 (5)	0.0083 (5)	0.0007 (6)
C2A	0.0281 (6)	0.0246 (7)	0.0240 (7)	0.0012 (5)	0.0097 (5)	0.0007 (5)
C3A	0.0254 (6)	0.0298 (7)	0.0288 (7)	−0.0006 (6)	0.0106 (5)	−0.0019 (6)
C4A	0.0269 (6)	0.0286 (7)	0.0240 (6)	0.0037 (6)	0.0137 (5)	−0.0019 (5)
C5A	0.0317 (7)	0.0281 (7)	0.0327 (7)	−0.0012 (6)	0.0157 (6)	−0.0026 (6)
C6A	0.0374 (7)	0.0254 (7)	0.0325 (7)	0.0032 (6)	0.0176 (6)	0.0051 (6)
C7A	0.0314 (7)	0.0277 (7)	0.0245 (7)	0.0086 (6)	0.0142 (6)	0.0024 (5)
C8A	0.0377 (8)	0.0341 (8)	0.0301 (7)	0.0111 (7)	0.0137 (6)	0.0091 (6)
C9A	0.0339 (7)	0.0470 (9)	0.0279 (7)	0.0123 (7)	0.0047 (6)	0.0058 (7)
C10A	0.0296 (7)	0.0398 (9)	0.0354 (8)	0.0012 (6)	0.0067 (6)	−0.0025 (7)
C11A	0.0319 (7)	0.0287 (7)	0.0308 (7)	0.0038 (6)	0.0109 (6)	0.0022 (6)
C12A	0.0284 (6)	0.0259 (7)	0.0236 (6)	0.0061 (5)	0.0129 (5)	0.0002 (5)
C13A	0.0288 (7)	0.0256 (7)	0.0226 (6)	0.0051 (6)	0.0121 (5)	0.0039 (5)
C14A	0.0227 (6)	0.0321 (7)	0.0255 (7)	−0.0040 (6)	0.0085 (5)	0.0037 (6)
C15A	0.0335 (7)	0.0348 (8)	0.0273 (7)	−0.0019 (6)	0.0134 (6)	0.0036 (6)
C16A	0.0374 (8)	0.0508 (10)	0.0294 (8)	−0.0096 (7)	0.0159 (6)	−0.0042 (7)
C17A	0.0285 (7)	0.0790 (13)	0.0237 (7)	−0.0118 (8)	0.0083 (6)	0.0015 (8)
C18A	0.0243 (7)	0.0737 (12)	0.0314 (8)	0.0018 (7)	0.0076 (6)	0.0163 (8)
C19A	0.0205 (6)	0.0432 (9)	0.0327 (7)	−0.0008 (6)	0.0097 (6)	0.0087 (6)
C20A	0.0778 (12)	0.0473 (10)	0.0380 (9)	0.0246 (9)	0.0341 (9)	0.0059 (8)
O1B	0.0328 (5)	0.0364 (6)	0.0346 (6)	−0.0051 (5)	0.0088 (4)	−0.0119 (5)
O2B	0.0270 (5)	0.0447 (6)	0.0431 (6)	−0.0023 (4)	0.0156 (4)	−0.0194 (5)
N1B	0.0234 (6)	0.0434 (8)	0.0384 (7)	0.0017 (5)	0.0038 (5)	−0.0168 (6)
N2B	0.0217 (6)	0.0481 (8)	0.0415 (7)	0.0025 (5)	0.0054 (5)	−0.0238 (6)
C1B	0.0258 (6)	0.0315 (8)	0.0229 (7)	0.0026 (6)	0.0067 (5)	−0.0045 (6)
C2B	0.0232 (6)	0.0295 (7)	0.0289 (7)	0.0012 (5)	0.0085 (5)	−0.0054 (6)
C3B	0.0254 (7)	0.0395 (8)	0.0250 (7)	0.0027 (6)	0.0079 (6)	−0.0047 (6)
C4B	0.0235 (6)	0.0317 (7)	0.0228 (6)	0.0033 (6)	0.0105 (5)	−0.0058 (6)
C5B	0.0353 (7)	0.0260 (7)	0.0293 (7)	0.0046 (6)	0.0123 (6)	−0.0001 (6)
C6B	0.0355 (7)	0.0238 (7)	0.0350 (8)	−0.0037 (6)	0.0160 (6)	−0.0033 (6)
C7B	0.0268 (6)	0.0262 (7)	0.0272 (7)	−0.0020 (5)	0.0135 (5)	−0.0076 (5)
C8B	0.0286 (7)	0.0359 (8)	0.0365 (8)	−0.0088 (6)	0.0116 (6)	−0.0122 (7)
C9B	0.0257 (7)	0.0480 (9)	0.0314 (8)	0.0018 (7)	0.0044 (6)	−0.0130 (7)
C10B	0.0352 (7)	0.0426 (9)	0.0252 (7)	0.0086 (7)	0.0087 (6)	−0.0014 (6)
C11B	0.0324 (7)	0.0308 (8)	0.0277 (7)	0.0012 (6)	0.0140 (6)	−0.0006 (6)
C12B	0.0244 (6)	0.0264 (7)	0.0225 (6)	−0.0006 (5)	0.0121 (5)	−0.0056 (5)
C13B	0.0241 (6)	0.0278 (7)	0.0264 (7)	−0.0042 (5)	0.0132 (5)	−0.0055 (6)
C14B	0.0242 (6)	0.0311 (7)	0.0221 (6)	−0.0002 (6)	0.0073 (5)	0.0003 (5)
C15B	0.0266 (7)	0.0350 (8)	0.0248 (7)	−0.0008 (6)	0.0088 (5)	−0.0012 (6)
C16B	0.0267 (7)	0.0436 (9)	0.0306 (7)	0.0030 (6)	0.0133 (6)	0.0022 (6)
C17B	0.0251 (7)	0.0525 (10)	0.0311 (7)	−0.0027 (7)	0.0101 (6)	0.0048 (7)
C18B	0.0294 (7)	0.0445 (9)	0.0270 (7)	−0.0084 (6)	0.0069 (6)	−0.0001 (6)
C19B	0.0317 (7)	0.0327 (8)	0.0233 (7)	−0.0017 (6)	0.0093 (6)	0.0013 (6)
C20B	0.0360 (8)	0.0412 (9)	0.0420 (9)	0.0031 (7)	0.0198 (7)	−0.0120 (7)

### Geometric parameters (Å, º)

**Table:**

O1A—C19A	1.3539 (19)	O1B—C19B	1.3536 (17)
O1A—H1OA	0.97 (2)	O1B—H1OB	0.91 (2)
O2A—C15A	1.3582 (17)	O2B—C15B	1.3648 (17)
O2A—C20A	1.4313 (18)	O2B—C20B	1.4269 (17)
N1A—C1A	1.2910 (18)	N1B—C1B	1.2915 (18)
N1A—N2A	1.4059 (16)	N1B—N2B	1.4016 (16)
N2A—C3A	1.4745 (18)	N2B—C3B	1.469 (2)
N2A—H2NA	0.914 (18)	N2B—H2NB	0.911 (19)
C1A—C14A	1.4664 (18)	C1B—C14B	1.4699 (18)
C1A—C2A	1.5091 (18)	C1B—C2B	1.5121 (18)
C2A—C3A	1.5338 (18)	C2B—C3B	1.5354 (18)
C2A—H2AA	0.9900	C2B—H2BA	0.9900
C2A—H2AB	0.9900	C2B—H2BB	0.9900
C3A—C4A	1.5104 (18)	C3B—C4B	1.5155 (18)
C3A—H3AA	1.0000	C3B—H3BA	1.0000
C4A—C13A	1.3742 (19)	C4B—C13B	1.3706 (19)
C4A—C5A	1.4104 (19)	C4B—C5B	1.412 (2)
C5A—C6A	1.368 (2)	C5B—C6B	1.369 (2)
C5A—H5AA	0.9500	C5B—H5BA	0.9500
C6A—C7A	1.416 (2)	C6B—C7B	1.413 (2)
C6A—H6AA	0.9500	C6B—H6BA	0.9500
C7A—C8A	1.4210 (19)	C7B—C12B	1.4216 (19)
C7A—C12A	1.4218 (19)	C7B—C8B	1.4239 (19)
C8A—C9A	1.361 (2)	C8B—C9B	1.366 (2)
C8A—H8AA	0.9500	C8B—H8BA	0.9500
C9A—C10A	1.405 (2)	C9B—C10B	1.404 (2)
C9A—H9AA	0.9500	C9B—H9BA	0.9500
C10A—C11A	1.372 (2)	C10B—C11B	1.368 (2)
C10A—H10A	0.9500	C10B—H10B	0.9500
C11A—C12A	1.4181 (19)	C11B—C12B	1.4128 (19)
C11A—H11A	0.9500	C11B—H11B	0.9500
C12A—C13A	1.4200 (18)	C12B—C13B	1.4211 (18)
C13A—H13A	0.9500	C13B—H13B	0.9500
C14A—C19A	1.4070 (19)	C14B—C15B	1.4096 (19)
C14A—C15A	1.408 (2)	C14B—C19B	1.4159 (19)
C15A—C16A	1.390 (2)	C15B—C16B	1.3909 (18)
C16A—C17A	1.381 (2)	C16B—C17B	1.387 (2)
C16A—H16A	0.9500	C16B—H16B	0.9500
C17A—C18A	1.371 (3)	C17B—C18B	1.372 (2)
C17A—H17A	0.9500	C17B—H17B	0.9500
C18A—C19A	1.392 (2)	C18B—C19B	1.3936 (19)
C18A—H18A	0.9500	C18B—H18B	0.9500
C20A—H20A	0.9800	C20B—H20D	0.9800
C20A—H20B	0.9800	C20B—H20E	0.9800
C20A—H20C	0.9800	C20B—H20F	0.9800
			
C19A—O1A—H1OA	108.1 (13)	C19B—O1B—H1OB	107.5 (14)
C15A—O2A—C20A	117.08 (12)	C15B—O2B—C20B	117.68 (10)
C1A—N1A—N2A	109.97 (11)	C1B—N1B—N2B	110.08 (11)
N1A—N2A—C3A	107.15 (11)	N1B—N2B—C3B	107.86 (11)
N1A—N2A—H2NA	106.4 (11)	N1B—N2B—H2NB	108.9 (11)
C3A—N2A—H2NA	114.3 (11)	C3B—N2B—H2NB	114.9 (12)
N1A—C1A—C14A	119.87 (12)	N1B—C1B—C14B	119.81 (12)
N1A—C1A—C2A	111.55 (11)	N1B—C1B—C2B	111.08 (11)
C14A—C1A—C2A	128.58 (12)	C14B—C1B—C2B	129.05 (11)
C1A—C2A—C3A	100.66 (10)	C1B—C2B—C3B	101.06 (10)
C1A—C2A—H2AA	111.6	C1B—C2B—H2BA	111.6
C3A—C2A—H2AA	111.6	C3B—C2B—H2BA	111.6
C1A—C2A—H2AB	111.6	C1B—C2B—H2BB	111.6
C3A—C2A—H2AB	111.6	C3B—C2B—H2BB	111.6
H2AA—C2A—H2AB	109.4	H2BA—C2B—H2BB	109.4
N2A—C3A—C4A	113.94 (11)	N2B—C3B—C4B	111.84 (11)
N2A—C3A—C2A	101.37 (11)	N2B—C3B—C2B	101.06 (11)
C4A—C3A—C2A	114.39 (11)	C4B—C3B—C2B	115.47 (11)
N2A—C3A—H3AA	108.9	N2B—C3B—H3BA	109.4
C4A—C3A—H3AA	108.9	C4B—C3B—H3BA	109.4
C2A—C3A—H3AA	108.9	C2B—C3B—H3BA	109.4
C13A—C4A—C5A	118.88 (12)	C13B—C4B—C5B	119.33 (12)
C13A—C4A—C3A	122.98 (12)	C13B—C4B—C3B	121.31 (12)
C5A—C4A—C3A	118.12 (12)	C5B—C4B—C3B	119.36 (12)
C6A—C5A—C4A	121.26 (13)	C6B—C5B—C4B	120.80 (13)
C6A—C5A—H5AA	119.4	C6B—C5B—H5BA	119.6
C4A—C5A—H5AA	119.4	C4B—C5B—H5BA	119.6
C5A—C6A—C7A	120.92 (13)	C5B—C6B—C7B	121.10 (13)
C5A—C6A—H6AA	119.5	C5B—C6B—H6BA	119.5
C7A—C6A—H6AA	119.5	C7B—C6B—H6BA	119.5
C6A—C7A—C8A	122.70 (13)	C6B—C7B—C12B	118.54 (12)
C6A—C7A—C12A	118.45 (12)	C6B—C7B—C8B	122.99 (13)
C8A—C7A—C12A	118.83 (13)	C12B—C7B—C8B	118.46 (13)
C9A—C8A—C7A	120.96 (13)	C9B—C8B—C7B	120.95 (14)
C9A—C8A—H8AA	119.5	C9B—C8B—H8BA	119.5
C7A—C8A—H8AA	119.5	C7B—C8B—H8BA	119.5
C8A—C9A—C10A	120.46 (13)	C8B—C9B—C10B	120.43 (13)
C8A—C9A—H9AA	119.8	C8B—C9B—H9BA	119.8
C10A—C9A—H9AA	119.8	C10B—C9B—H9BA	119.8
C11A—C10A—C9A	120.27 (14)	C11B—C10B—C9B	120.01 (14)
C11A—C10A—H10A	119.9	C11B—C10B—H10B	120.0
C9A—C10A—H10A	119.9	C9B—C10B—H10B	120.0
C10A—C11A—C12A	120.87 (13)	C10B—C11B—C12B	121.32 (13)
C10A—C11A—H11A	119.6	C10B—C11B—H11B	119.3
C12A—C11A—H11A	119.6	C12B—C11B—H11B	119.3
C11A—C12A—C13A	122.43 (12)	C11B—C12B—C13B	122.25 (12)
C11A—C12A—C7A	118.60 (12)	C11B—C12B—C7B	118.81 (12)
C13A—C12A—C7A	118.97 (12)	C13B—C12B—C7B	118.94 (12)
C4A—C13A—C12A	121.50 (12)	C4B—C13B—C12B	121.28 (12)
C4A—C13A—H13A	119.2	C4B—C13B—H13B	119.4
C12A—C13A—H13A	119.2	C12B—C13B—H13B	119.4
C19A—C14A—C15A	117.46 (13)	C15B—C14B—C19B	117.35 (12)
C19A—C14A—C1A	120.57 (12)	C15B—C14B—C1B	122.59 (12)
C15A—C14A—C1A	121.95 (12)	C19B—C14B—C1B	120.05 (12)
O2A—C15A—C16A	123.43 (13)	O2B—C15B—C16B	123.04 (12)
O2A—C15A—C14A	115.27 (12)	O2B—C15B—C14B	115.96 (11)
C16A—C15A—C14A	121.28 (14)	C16B—C15B—C14B	120.99 (13)
C17A—C16A—C15A	119.08 (15)	C17B—C16B—C15B	119.57 (13)
C17A—C16A—H16A	120.5	C17B—C16B—H16B	120.2
C15A—C16A—H16A	120.5	C15B—C16B—H16B	120.2
C18A—C17A—C16A	121.53 (14)	C18B—C17B—C16B	121.40 (13)
C18A—C17A—H17A	119.2	C18B—C17B—H17B	119.3
C16A—C17A—H17A	119.2	C16B—C17B—H17B	119.3
C17A—C18A—C19A	119.57 (14)	C17B—C18B—C19B	119.32 (14)
C17A—C18A—H18A	120.2	C17B—C18B—H18B	120.3
C19A—C18A—H18A	120.2	C19B—C18B—H18B	120.3
O1A—C19A—C18A	117.18 (13)	O1B—C19B—C18B	117.24 (12)
O1A—C19A—C14A	121.87 (13)	O1B—C19B—C14B	121.46 (12)
C18A—C19A—C14A	120.94 (14)	C18B—C19B—C14B	121.30 (13)
O2A—C20A—H20A	109.5	O2B—C20B—H20D	109.5
O2A—C20A—H20B	109.5	O2B—C20B—H20E	109.5
H20A—C20A—H20B	109.5	H20D—C20B—H20E	109.5
O2A—C20A—H20C	109.5	O2B—C20B—H20F	109.5
H20A—C20A—H20C	109.5	H20D—C20B—H20F	109.5
H20B—C20A—H20C	109.5	H20E—C20B—H20F	109.5

### Hydrogen-bond geometry (Å, º)

**Table:**

*D*—H···*A*	*D*—H	H···*A*	*D*···*A*	*D*—H···*A*
O1*A*—H1*OA*···N1*A*	0.97 (2)	1.71 (2)	2.5754 (15)	145 (2)
O1*B*—H1*OB*···N1*B*	0.91 (2)	1.71 (2)	2.5377 (15)	149 (2)
N2*A*—H2*NA*···O1*A*^i^	0.914 (18)	2.234 (18)	3.0470 (16)	147.7 (15)
N2*B*—H2*NB*···O1*B*^ii^	0.911 (19)	2.131 (19)	2.9787 (16)	154.4 (15)

Symmetry codes: (i) −*x*+1, −*y*+1, −*z*+1; (ii) −*x*, −*y*−2, −*z*+1.
